# Clinical Characteristics of Coccidioidomycosis in Patients with Hepatitis C-Associated Cirrhosis

**DOI:** 10.3390/microorganisms14102296

**Published:** 2026-10-09

**Authors:** Rayne Shepard, Nandini Nukala, Marina Roytman, Seema Policepatil, Geetha Sivasubramanian

**Affiliations:** 1 Clinical Research Center, University of California, San Francisco, Fresno, CA 93701, USA; rayne.shepard7@gmail.com; 2 Department of Biological Sciences, Nova Southeastern University, Ft. Lauderdale, FL 33328, USA; nandini.v.nukala@gmail.com; 3 Division of Gastroenterology and Hepatology, University of California, San Francisco, Fresno, CA 93701, USA; marina.roytman@ucsf.edu; 4 Department of Internal Medicine, University of California, San Francisco, Fresno, CA 93701, USA; seema.policepatil@ucsf.edu; 5 Division of Infectious Diseases, University of California, San Francisco, Fresno, CA 93701, USA

**Keywords:** coccidioidomycosis, hepatitis C virus, end-stage liver disease, cirrhosis, disseminated fungal infection

## Abstract

Coccidioidomycosis is a fungal infection with increased morbidity in immunocompromised populations. Patients with hepatitis C virus (HCV)-associated cirrhosis may be at increased risk because of cirrhosis-associated immune dysfunction; however, data specifically describing this intersection are limited. We conducted a retrospective chart review of patients diagnosed with coccidioidomycosis between 2010 and 2025 at a tertiary care center in Central California who had a pre-existing or concurrent diagnosis of HCV-associated cirrhosis. Demographic characteristics, clinical presentation, disease course, treatment history for HCV and coccidioidomycosis, and outcomes were reviewed. Ten patients met the inclusion criteria. Most patients were symptomatic at presentation, and half required hospitalization. Complicated pulmonary disease was common, and extrapulmonary dissemination occurred in three patients, involving the central nervous system, bone, and skin and soft tissue. Among patients who relapsed, none had received HCV treatment before the coccidioidomycosis diagnosis. Only two patients achieved sustained virologic response (SVR) before their coccidioidomycosis diagnosis, and neither relapsed. As a descriptive case series without a comparator group, this study characterizes the clinical course in this subgroup but cannot establish whether HCV-associated cirrhosis is associated with greater severity, dissemination, relapse, or mortality. These observations are hypothesis-generating and require prospective, comparative studies in larger cohorts.

## 1. Introduction

Coccidioidomycosis, also known as Valley Fever, is a fungal infection caused by inhalation of spores from the dimorphic fungi *Coccidioides immitis* and *Coccidioides posadasii* [1,2]. The fungi are largely found in the southwestern United States and northern Mexico but have recently been observed in the Pacific Northwest and in Central and South America [3,4,5]. Clinical manifestations range from asymptomatic infection to symptomatic pulmonary disease, with a minority progressing to extrapulmonary dissemination [6]. Disseminated disease occurs most prominently in patients with pre-existing immunocompromising conditions such as cancer chemotherapy, Human Immunodeficiency Virus (HIV), and organ transplantation [7].

Cirrhosis has emerged as a risk factor for disseminated coccidioidomycosis, likely due to the immune dysfunction associated with advanced liver disease [8]. The extent to which specific etiologies of cirrhosis modify this risk remains an open question. Among the various etiologies, Hepatitis C virus (HCV) infection remains one of the most common causes of cirrhosis. In Central California, where both coccidioidomycosis and HCV infection are highly prevalent, these two conditions often coexist.

HCV causes progressive liver damage, leading from fibrosis to cirrhosis if untreated [9,10]. The virus has six major clinical genotypes, with genotype 3 associated with higher rates of hepatic steatosis, cirrhotic progression, and hepatocellular carcinoma; this genetic diversity has precluded vaccine development [11]. While some individuals spontaneously clear the infection, many develop chronic hepatitis C, which correlates with increased hospitalization, mortality, and comorbidities [12,13]. Chronic infection drives progressive fibrosis and cirrhosis-associated immune dysfunction, characterized by impaired innate and adaptive immune responses [10,14,15]. This immune dysregulation increases susceptibility to bacterial and fungal infections, including endemic mycoses such as coccidioidomycosis [16,17]. Consequently, patients with untreated HCV and advanced liver disease may be particularly vulnerable to severe or disseminated fungal infections [18]. Fungal infections occur in 10–13% of patients with cirrhosis, and dual bacterial-fungal infections are associated with lower survival than bacterial infections alone [19,20,21].

Despite this biological plausibility, the available literature does not specifically stratify outcomes by the etiology of cirrhosis. Existing studies include mixed etiologies, with alcohol-related liver disease being the most common in the Nukala cohort [8]. Here, we describe a retrospective case series focusing specifically on the intersection of HCV-associated cirrhosis and coccidioidomycosis.

## 2. Materials and Methods

We conducted a retrospective single-center descriptive case series of patients diagnosed with coccidioidomycosis between January 2010 and January 2025 who had a pre-existing or concurrent diagnosis of HCV-associated cirrhosis and/or end-stage liver disease (ESLD) at Community Regional Medical Center (CRMC) in Fresno, CA. This study was approved by the CRMC Institutional Review Board. Informed consent was waived due to the retrospective nature of the study.

CRMC is a 750-bed tertiary care center located in the San Joaquin Valley and serves as the primary teaching hospital for the University of California, San Francisco, Fresno. It is the main referral center for the surrounding cities and counties in Central California, a region endemic for coccidioidomycosis.

### 2.1. Data Collection

Patients were identified using International Classification of Diseases, Ninth Revision (ICD-9) and Tenth Revision (ICD-10) diagnostic codes for coccidioidomycosis (ICD-9: 114.x; ICD-10: B38.x), chronic hepatitis C (ICD-9: 070.44, 070.54, 070.70, 070.71; ICD-10: B18.2), cirrhosis (ICD-9: 571.5; ICD-10: K74.x), and end-stage liver disease (ICD-9: 572.8; ICD-10: K72.90) [22]. ICD coding was the sole case-identification source. Every identified record then underwent individual chart review to confirm both diagnoses: coccidioidomycosis required microbiologic, serologic, or histopathologic confirmation, and HCV was required to be the documented etiology of cirrhosis/ESLD. This chart-review step served to validate the diagnostic coding and to minimize misclassification. All eligible patients identified through the ICD-based search during the study period were included.

Collected demographic variables included age, sex, race, and ethnicity. Chart review confirmed the presence of a prior or concurrent diagnosis of HCV-associated cirrhosis/ESLD at the time of coccidioidomycosis diagnosis.

For HCV infection, collected data included the date of diagnosis, the documented suspected route of transmission, and viral genotype. We also recorded whether patients achieved sustained virologic response (SVR), the antiviral regimen used, and whether treatment occurred before or after the diagnosis of coccidioidomycosis. The interval between HCV treatment and the diagnosis of coccidioidomycosis was documented when applicable.

### 2.2. Inclusion and Exclusion Criteria

Patients were included if they had a confirmed diagnosis of coccidioidomycosis with concurrent or pre-existing HCV-associated cirrhosis and/or ESLD. Patients were excluded if medical records lacked sufficient documentation to confirm HCV etiology of cirrhosis or if a diagnosis of coccidioidomycosis could not be verified by microbiologic, serologic, or histopathologic evidence.

### 2.3. Definitions

End-stage liver disease: Presence of cirrhosis with either listing for liver transplantation or clinical decompensation, including ascites requiring diuretics or paracentesis, grade 3–4 esophageal variceal bleeding, hepatic encephalopathy, spontaneous bacterial peritonitis, hepatorenal syndrome, model for end-stage liver disease (MELD) 3.0 score ≥ 15, or jaundice with total bilirubin ≥ 3 mg/dL.

Cirrhosis: Diagnosis of hepatic cirrhosis established by liver histology, or by radiologic (ultrasound, CT, or MRI), elastographic, or endoscopic findings consistent with cirrhosis, and/or clinical and laboratory evidence of cirrhosis documented in the medical record.

HCV infection: Positive hepatitis C antibody with confirmatory quantitative RNA testing.

Sustained virologic response (SVR): undetectable serum HCV RNA at least 12 weeks after completion of antiviral therapy.

Coccidioidomycosis: Confirmed infection based on positive serology (enzyme immunoassay, immunodiffusion, or complement fixation), culture, or histopathologic identification of *Coccidioides* species.

Disseminated coccidioidomycosis: Evidence of disease outside the thoracic cavity with confirmation through microbiologic or histopathologic samples at the affected sites.

Complicated pulmonary disease: Progressive pulmonary infection such as pneumonia, fibro-cavitary disease, empyema, or ruptured cavity.

Resolution of coccidioidomycosis: Treating-provider documentation of clinical resolution with discontinuation of antifungal therapy, supported by one or more of the following when available: absence of symptoms, declining or stable serologic titers, or stable or improving radiologic findings.

Relapse of coccidioidomycosis: Treating-provider documentation of recurrent coccidioidomycosis after initial clinical improvement or treatment completion, supported by one or more of the following when available: recurrent symptoms, rising serologic titers, or new radiologic findings, with or without resumption of antifungal therapy.

## 3. Results

ICD-9/ICD-10 coding identified 46 patients with cirrhosis and/or ESLD and confirmed coccidioidomycosis during the study period. On individual chart review, 11 had HCV documented as the etiology of cirrhosis/ESLD; one was excluded because the record lacked sufficient documentation to confirm HCV etiology, leaving 10 patients who met all inclusion criteria. The cohort comprised six males and four females.

The median age at coccidioidomycosis diagnosis was 49.5 years (range, 33–65 years) (Table 1). Seven patients were identified as Hispanic and three as non-Hispanic. All patients were residents of Central California and resided in rural areas of the San Joaquin Valley at the time of diagnosis. Potential occupational or environmental exposures were identified in several patients, including truck driving (*n* = 2), construction work (*n* = 1), a history of incarceration (*n* = 1), and frequent outdoor gardening (*n* = 1).

All patients had a prior diagnosis of HCV infection at the time of coccidioidomycosis diagnosis, with a median interval of approximately 12 years between diagnoses (range 2 to >25 years). HCV genotype 1a was most common (*n* = 6), followed by genotype 3a (*n* = 3); genotype was unknown in one patient. Intravenous drug use was the most frequently documented transmission route. All patients had HCV-associated cirrhosis; seven had decompensated cirrhosis at the time of coccidioidomycosis diagnosis. The median MELD score was 16.5 (range, 10–41). Two patients had completed HCV treatment prior to coccidioidomycosis diagnosis, approximately 8 years and 10 months before the fungal infection, respectively; four achieved SVR after their fungal diagnosis, and four remained untreated at the time of chart review. Among the six treated patients, five received all-oral direct-acting antiviral (DAA) regimens (sofosbuvir/velpatasvir, ledipasvir/sofosbuvir, or sofosbuvir-based combinations), while one patient treated in 2011 had a regimen that could not be confirmed from the record. No interferon-based therapy was documented.

Nine of ten patients were symptomatic at presentation with coccidioidomycosis (Table 1). The most common presenting symptoms included cough (*n* = 7), dyspnea (*n* = 5), and fever (*n* = 4). The median symptom duration before diagnosis was 8 weeks (range, 1–48 weeks). Symptom duration before diagnosis was 1 week in each of the two patients with SVR prior to coccidioidomycosis diagnosis and a median of 9 weeks (range 2–48) in the remaining seven patients with available data. Chest imaging revealed pulmonary nodules (*n* = 7), cavitary lesions (*n* = 4), pleural effusions (*n* = 2), and consolidation (*n* = 3). Diagnosis was confirmed by histopathology (*n* = 4), serology (*n* = 8), and/or culture (*n* = 3). Complement fixation titers at diagnosis ranged from negative to 1:512. Seven patients developed complicated pulmonary coccidioidomycosis. Extrapulmonary dissemination occurred in three patients, involving the central nervous system (*n* = 2), skin and soft tissue (*n* = 3), and bone (*n* = 3), with some individuals having multiple sites of dissemination. Five patients required hospitalization for their fungal infection (Table 1).

All ten patients received antifungal therapy. Fluconazole was administered at an initial dose of 400 mg daily to most patients, with one patient treated at 200 mg and another at 600 mg daily. No patient developed documented azole hepatotoxicity, and none discontinued antifungal therapy because of hepatotoxicity; non-adherence was not documented in the available records, though adherence could not be systematically assessed given the retrospective design. The median duration of antifungal therapy was 31 weeks (range, 1–335 weeks) among the nine patients with available treatment data. The median follow-up duration after coccidioidomycosis diagnosis was 251 weeks (range, 2–610 weeks). Patients continued longitudinal follow-up after clinical resolution with periodic clinical evaluation, serologic testing, and radiographic imaging to monitor disease recurrence. Resolution of coccidioidomycosis was achieved in nine patients, with a median time from diagnosis to resolution of 57 weeks (range, 19–335 weeks). One patient did not achieve resolution. Relapse occurred in five patients. Among these patients, the median time to relapse from clinical resolution was 152 weeks (range, 9–241 weeks). Of the five patients who relapsed, one was untreated for HCV, and four achieved SVR only after their coccidioidomycosis diagnosis; neither of the two patients with SVR before diagnosis relapsed.

All-cause mortality, recorded as a follow-up observation rather than a predefined endpoint, occurred in five of ten patients during longitudinal follow-up, at 2, 42, 110, 196, and 609 weeks from coccidioidomycosis diagnosis. Cause-specific mortality could not be determined, as autopsy reports and death-certificate data were not available; deaths are therefore reported as all-cause only and cannot be attributed to coccidioidomycosis, as they may reflect underlying end-stage liver disease or other comorbidities.

## 4. Discussion

To our knowledge, this retrospective case series is the first to examine coccidioidomycosis in patients with HCV-associated cirrhosis/ESLD specifically. Our findings highlight the significant morbidity associated with this intersection, including high rates of complicated pulmonary disease, extrapulmonary dissemination, and relapse.

Most patients in this cohort were symptomatic at the time of diagnosis, and half required hospitalization. The median age at diagnosis was 49.5 years (range, 33–65 years), and the median MELD 3.0 score at presentation was 16.5. Two incidental observations in the two patients who achieved SVR before their coccidioidomycosis diagnosis, namely shorter symptom duration before diagnosis and absence of relapse, are reported as anecdotal findings only. Against five relapses overall, the small numbers preclude meaningful comparison, and no inference about the effect of HCV treatment or its timing on the clinical course of coccidioidomycosis can be drawn. Possible explanations, such as improved immune function after viral eradication or greater engagement with care, facilitating earlier diagnosis [23,24], or better hepatic tolerance of antifungal therapy with reduced hepatotoxicity [25,26,27], remain speculative and require validation in larger cohorts. Although five of ten patients died during follow-up, these were all-cause deaths; in the absence of autopsy or death-certificate data, and given the competing risks from end-stage liver disease and other comorbidities, mortality is not presented as an indicator of coccidioidomycosis severity.

Overall, the clinical course observed in this case series aligns with previously published literature describing increased severity of disseminated coccidioidomycosis in patients with multiple comorbid conditions, including hypertension and diabetes mellitus [28,29,30]. These findings further support the role of underlying systemic illness in modulating disease severity and outcomes in coccidioidomycosis. Prior series have characterized coccidioidomycosis in cirrhosis and ESLD of mixed etiologies, in which alcohol-related liver disease predominated and HCV represented a minority of cases. The present series builds on this work by isolating the HCV-associated subgroup, confirming that the high rates of complicated pulmonary disease, dissemination, hospitalization, and relapse described in mixed-etiology cohorts are also seen when HCV is the sole underlying etiology, while adding descriptive detail on HCV genotype, treatment status, and SVR timing not captured in those reports [8].

Patients with HCV-associated end-stage liver disease may be particularly susceptible to severe or disseminated coccidioidomycosis due to underlying immune dysregulation [31,32]. Although biologically plausible, this retrospective study was not designed to determine whether HCV-associated cirrhosis confers greater risk than cirrhosis of other etiologies. Cirrhosis is associated with impaired innate and adaptive immune responses, including reduced complement activity, altered macrophage function, and T-cell dysfunction, which may compromise host defense against fungal pathogens [33]. Beyond cirrhosis-associated immune dysfunction, chronic HCV infection itself impairs host immunity through multiple mechanisms [25,31,32]. Notably, a large Taiwanese cohort study found that patients with noncirrhotic HCV infection had a significantly increased risk of hospitalization for infection (adjusted HR 1.22), including mycotic infections, compared to uninfected controls, an association not observed with hepatitis B infection [34]. Studies of patients with end-stage liver disease and cryptococcal infection have similarly suggested that viral hepatitis etiologies, including HCV and hepatitis B virus, may confer additional risk compared to other causes of cirrhosis [17]. Whether HCV-specific immune perturbations contribute to the severity of coccidioidomycosis independently of cirrhosis stage warrants further investigation. It also remains uncertain whether viral eradication partially restores immune competence and hepatic function in this setting [31,32,33]. Whether these mechanisms contributed to the clinical observations in our cohort cannot be determined from this study.

The majority of patients in this cohort developed complicated pulmonary disease from coccidioidomycosis, including cavitary lesions, pleural effusions, and progressive pneumonia. Cirrhosis-related immune dysfunction may reduce containment of fungal infection within the lungs [35,36]. Advanced cirrhosis is also frequently complicated by hepatic hydrothorax [37,38,39]. However, the distinction between hepatic hydrothorax and coccidioidal pleural involvement was not systematically assessed in this series.

This study has several limitations. The retrospective design limits the ability to establish causal relationships between hepatitis C treatment status and coccidioidomycosis outcomes. This case series also lacked a comparator group; neither patients with non-HCV cirrhosis nor patients with non-cirrhotic HCV infection were included. It therefore cannot determine whether HCV-associated cirrhosis confers a greater risk of severe or disseminated coccidioidomycosis than cirrhosis of other etiologies, or whether HCV infection increases risk independent of cirrhosis. The observed rates of complicated pulmonary disease, dissemination, hospitalization, relapse, and death should accordingly be read descriptively rather than as measures of incremental risk. Most patients also had decompensated cirrhosis and a high burden of comorbidities, including diabetes and renal disease, which may independently influence infection severity, hospitalization, relapse, and mortality, and cannot be disentangled from HCV treatment status in a cohort of this size. The small sample size and single-center setting further limit meaningful statistical comparison and the generalizability of findings to other populations or non-endemic regions. Case identification based on ICD coding may have resulted in missed cases or misclassification. Although case capture spanned 2010 to 2025, a period over which HCV therapy and diagnostic practices evolved substantially, nearly all treated patients received contemporary all-oral DAA regimens, limiting treatment-era heterogeneity within the cohort. Variability in antifungal regimens and follow-up duration may also have influenced observed outcomes, and serologic titers and clinical documentation were not uniformly available for all patients, raising the possibility of under- or over-estimation of disease severity or relapse. Despite these limitations, this case series highlights clinically relevant patterns that warrant further investigation.

The findings of this study suggest that clinicians practicing in coccidioidomycosis-endemic regions should maintain a high index of suspicion for complicated pulmonary and disseminated disease in patients with HCV-associated end-stage liver disease presenting with systemic or pulmonary symptoms. Early diagnostic evaluation and prompt initiation of antifungal therapy may be particularly important in this population. Because this cohort was drawn from a rural, predominantly Hispanic population in a highly endemic region, the demographic and exposure profile reflects local epidemiology, and the observed frequencies of complicated and disseminated disease should not be assumed to transfer to lower-exposure settings. The underlying host vulnerability conferred by HCV-associated cirrhosis, however, is not region-dependent; in less endemic areas, coccidioidomycosis in these patients would be encountered chiefly in those with a relevant travel or residence history, in whom a similar index of suspicion is warranted when compatible pulmonary or systemic symptoms arise. Whether timely treatment of hepatitis C influences the clinical course of coccidioidomycosis remains uncertain and warrants further investigation. Future studies with larger cohorts are needed to clarify the relationship between hepatitis C treatment timing and coccidioidomycosis outcomes, to determine whether HCV-specific immune dysfunction contributes to disease severity independent of cirrhosis stage, and to stratify outcomes by cirrhosis etiology. Such data would inform screening and management strategies in high-risk patients.

## 5. Conclusions

Coccidioidomycosis in this group of patients with HCV-associated cirrhosis was often severe. Complicated pulmonary disease and dissemination were common; many patients were hospitalized, and the clinical course was prolonged, with relapse in five of ten patients. Because this is a small case series with no comparator group, we cannot say whether these outcomes are particular to HCV-associated cirrhosis or reflect advanced liver disease more broadly. What the series does show is that this is a high-risk, understudied population that warrants a high index of suspicion, and the questions it raises, including whether the timing of HCV treatment matters, will need larger comparative studies to answer.

## Figures and Tables

**Table 1:** Patient-level clinical characteristics, treatment, and outcomes of patients with coccidioidomycosis and HCV-associated cirrhosis/ESLD (*n* = 10).

	P1	P2	P3	P4	P5	P6	P7	P8	P9	P10
Demographics										
Age (years)	33	51	48	58	63	49	65	47	61	48
Sex	Male	Female	Female	Female	Male	Female	Male	Male	Male	Male
Ethnicity	Hispanic	Non-Hispanic	Hispanic	Non-Hispanic	Non-Hispanic	Hispanic	Hispanic	Hispanic	Hispanic	Hispanic
Comorbidities (non-hepatic)	Psoriasis	None	DM	DM	DM, CKD, COPD	None	DM, CKD	DM	DM	None
Hepatitis C/liver disease										
Decompensated cirrhosis	Yes	Yes	No	No	Yes	Yes	Yes	Yes	Yes	No
MELD	17	14	10	10	16	17	41	31	22	15
Genotype	1a	3a	1a	1a	1a	1a	Unknown	3a	3a	1a
Viral load (IU/mL)	116,906	1,772,243	Unknown	465,054	609,615	2,946,930	61,600,000	Unknown	1,950,000	165,846
SVR achieved	No	No	Yes	Yes	Yes	No	No	Yes	Yes	Yes
Treatment timing vs. coccidioidomycosis diagnosis	Untreated	Untreated	Before	After	After	Untreated	Untreated	Before	After	After
Presentation										
Symptomatic	Yes	Yes	Yes	Yes	Yes	No	Yes	Yes	Yes	Yes
Symptoms	Cough, fever, pleuritic chest pain	Cough, fever, chills	Hemoptysis, cough, dyspnea	Cough, dyspnea	Fatigue, dizziness, weight loss, LAD	None	Dyspnea, cough	Chest pain, cough, fever, dyspnea	Dyspnea	Weight loss, night sweats, cough, skin lesions, fever
Symptom duration (weeks)	9	48	1	8	20	N/A	2	1	20	8
Pulmonary findings	Nodules, cavitary	Nodules	Cavitary	Nodule	Cavitary, consolidation, nodules	Nodules	Effusion, consolidation	Effusion, consolidation	Nodules	Nodules, cavitary
Diagnostic method	Serology	Pathology	Serology	Pathology	Serology	Serology, Pathology	Serology, culture	Serology	Serology, culture	Serology, pathology, culture
CF titer at diagnosis	1:256	Unknown	1:2	Neg	1:64	Equivocal	1:512	Neg	1:16	1:128
Complicated pulmonary disease	Yes	Yes	Yes	No	Yes	No	Yes	Yes	No	Yes
Disseminated disease	Yes	No	No	No	Yes	No	No	No	No	Yes
Sites of dissemination	CNS, bone, skin, synovial	—	—	—	Soft tissue, bone	—	—	—	—	Bone, CNS, skin
Treatment and outcomes										
Hospitalized for coccidioidomycosis	Yes	No	Yes	No	No	No	Yes	Yes	No	Yes
Antifungal regimen	Fluconazole	Undocumented	Fluconazole	Fluconazole	Fluconazole	Fluconazole	Fluconazole	Fluconazole	Fluconazole	Itraconazole
Antifungal duration (weeks)	31	Undocumented	54	131	335	22	1	18	15	276
Resolution	Yes	Yes	Yes	Yes	Yes	Yes	No	Yes	Yes	Yes
Time to resolution (weeks)	31	33	57	131	335	25	—	19	140	276
Relapse	Yes	No	No	Yes	Yes	No	No	No	Yes	Yes
Time to relapse (weeks)	9	—	—	152	241	—	—	—	210	35
Follow-up (weeks)	42	196	305	430	609	110	2	19	351	610
Death (all-cause)	Yes	Yes	No	No	Yes	Yes	Yes	No	No	No
Time to death (weeks)	42	196	—	—	609	110	2	—	—	—

Abbreviations: CF, complement fixation; CKD, chronic kidney disease; CNS, central nervous system; COPD, chronic obstructive pulmonary disease; DM, diabetes mellitus; ESLD, end-stage liver disease; HCV, hepatitis C virus; IU, international units; LAD, lymphadenopathy; MELD, Model for End-Stage Liver Disease; SVR, sustained virologic response; not applicable or absent values are shown as “—”.

## Data Availability

The data that support the findings of this study were collected and entered into REDCap and are stored on secure servers at the University of California, San Francisco, Fresno. Restrictions apply to the availability of these data, which were used under license for the current study, and are not publicly available. Data are, however, available from the authors upon reasonable request and with permission from the Community Health Systems Institutional Review Board.

## Abbreviations

The following abbreviations are used in this manuscript:

HCV	Hepatitis C virus
ESLD	End-stage liver disease
HIV	Human immunodeficiency virus
CRMC	Community Regional Medical Center
ICD	International Classification of Diseases
SVR	Sustained virologic response
MELD	Model for end-stage liver disease
RNA	Ribonucleic acid
IU	International unit
